# Combined Photosensitization and Vaccination Enable CD8 T-Cell Immunity and Tumor Suppression Independent of CD4 T-Cell Help

**DOI:** 10.3389/fimmu.2019.01548

**Published:** 2019-07-05

**Authors:** Eleni Maria Varypataki, Fabio Hasler, Ying Waeckerle-Men, Sarah Vogel-Kindgen, Anders Høgset, Thomas M. Kündig, Bruno Gander, Cornelia Halin, Pål Johansen

**Affiliations:** ^1^Department of Dermatology, University of Zurich, Zurich, Switzerland; ^2^Institute of Pharmaceutical Sciences, ETH Zurich, Zurich, Switzerland; ^3^PCI Biotech AS, Oslo, Norway; ^4^Department of Dermatology, University Hospital Zurich, Zurich, Switzerland

**Keywords:** vaccine, photochemical internalization, cancer immunotherapy, melanoma, cytotoxic T cells, T-helper cells

## Abstract

Cytotoxic T lymphocytes (CTLs) are key players in fighting cancer, and their induction is a major focus in the design of therapeutic vaccines. Yet, therapeutic vaccine efficacy is limited, in part due to the suboptimal vaccine processing by antigen-presenting cells (APCs). Such processing typically takes place via the MHC class II pathway for CD4 T-cell activation and MHC class I pathway for activation of CD8 CTLs. We show that a combination of skin photochemical treatment and immunization, so-called photochemical internalization (PCI) facilitated CTL activation due to the photochemical adjuvant effect induced by photosensitizer, oxygen, and light. Mice were immunized intradermally with antigen and photosensitizer, followed by controlled light exposure. PCI-treated mice showed strong activation of CD8 T cells, with improved IFN-γ production and cytotoxicity, as compared to mice immunized without parallel PCI treatment. Surprisingly, the CD8 T-cell effector functions were not impaired in MHC class II- or CD4 T-cell-deficient mice. Moreover, PCI-based vaccination caused tumor regression independent of MHC class II or CD4 T cells presence in melanoma bearing mice. Together, the data demonstrate that PCI can act as a powerful adjuvant in cancer vaccines, even in hosts with impaired T-helper functions.

## Introduction

Therapeutic cancer vaccines, consisting of well-defined antigens, and carriers or adjuvants, aim at inducing cytotoxic T cells (CTLs) specific for the tumor (1). The CTL activation is primarily mediated by special subsets of dendritic cells (DCs), such as lymphoid CD8α DCs, skin CD103 DCs, or CD207 Langerhans cells (2, 3),which process and present antigens on MHC class I molecules. However, the processing of exogenous antigens by DCs or other antigen-presenting cells (APCs) typically leads to MHC class II antigen presentation to CD4 T cells and it is generally recognized that CD4 T-helper cells are required for optimal activation, expansion, homing, and maintenance of CTLs. A major challenge in the development of vaccines or antigen-delivery systems is to design preparations that are capable of delivering antigen to MHC class I molecules and, at the same time, ensuring sufficient help from CD4 T cells (1, 4). To this end, strategies that enable direct cytosolic delivery of the antigen either by translocation through the APC plasma membrane or by antigen uptake and subsequent endosomal escape have shown promising results (5–9).

Photochemical internalization (PCI) is a novel medical approach that enables cytosolic targeting of drugs in combination with a photosensitizer. Photosensitizer and drug are taken up by endocytosis and upon light activation, reactive oxygen species cause endosomal leakage, and translocation of the drug into the cytosol (10). When using the PCI technology with antigens, we have shown that in APCs PCI can mediate antigen delivery to cytosolic MHC class I for activation of CD8 T cells, at least *in vitro* and in transgenic mouse models (11–15). The goal of the current investigation was to study PCI-based immunization in wild type mice and in mouse tumor models. Moreover, since the hypothetic mode of action of PCI is based on the endosomal disruption and redirection of antigen presentation away from MHC class II, we also investigated if the removal of CD4 T-cell help would affect the stimulation of CTLs or otherwise the CTL function such as tumor suppression.

## Materials and Methods

### Mice

Female C57BL/6 (H-2K^b^) mice were purchased from Envigo (Horst, The Netherlands). Congenic CD45.1 (Ly5.1), MHC class II- and CD40L-deficient mice were provided through SwIMMR, the Swiss Immunological Mutant Mouse Repository (Schlieren, Switzerland), and bred at the animal facility at the *Biologische Zentrallabor*, University Hospital Zurich. All mice were kept under SPF conditions, and the experimental procedures were approved by the animal research ethics review board and the Swiss veterinary authorities (licenses ZH 200/2014 and ZH 52/2016).

### Cells

Mouse melanoma B16-F10 cells (ATCC CRL-6475) transfected for expression of OVA was kindly provided by Emmanuel Contassot (University of Zürich). The cells were cultured in RPMI-1640 medium (Sigma-Aldrich) supplemented with 8% FCS, 50 mg/ml Normocin (InvivoGen), 2 mM L-glutamine (Life Technologies, Zug, Switzerland), and 1 mg/mL G-418 (Life Technologies).

### Immunization and Photosensitization of Mice

Mice were immunized with chicken egg albumin (OVA) from Sigma-Aldrich (Buchs, Switzerland) and the photosensitizer tetraphenyl chlorine disulfonate (TPCS2a) was provided by PCI Biotech (Oslo, Norway). The mice received two intradermal immunizations of each 50 μl in the abdominal region, of the freshly prepared vaccines containing 100 μg of OVA and 50 μg of TPCS2a (in a total volume of 100 μl). Eighteen hours after the immunization, the mice were anesthetized with ketamine (75 mg/kg) and xylazine (12 mg/kg) by intraperitoneal injection and light-treated at 435 nm with LumiSource (PCI Biotech) for 6 min (4.86 J/cm^2^) as previously described (12). Two booster immunizations with 2 week intervals were typically given using the same dosing and illumination regimens as for priming. Blood and spleen were collected at different time points for further analysis of T- and B-cell responses.

As an alternative source of antigen, mice were immunized with lethally irradiated (50 Gy) OVA-expressing B16 melanoma cells (B16-OVA). After irradiation, the cells were washed, suspended in PBS, and 1 ×10^6^ cells were mixed with 50 μg of TPCS2a and 1 μg of poly(I:C) (InvivoGen; Toulouse, France) in 100 μl PBS and intradermally administered as described for the immunization with OVA. Light treatment and boosting immunizations were made as described for OVA.

### CD4 T-Cell Depletion and MHC Class-II Blocking

Depletion of CD4 T cells was done by intraperitoneal administration of a mixture of two monoclonal antibodies isolated from the YTS 191.1.2 (1 mg) and YTA 3.1.2 (1 mg) hybridoma cells (16). The antibodies were injected 1 day prior to and 4 days after each immunization. Alternatively, and to test whether CD4 T cells were important during antigen presentation or for some subsequent bystander effect, the antibodies were injected 4 days after immunization only. The depletion efficacy was verified by flow cytometry of the blood at different time points after injection.

For the inhibition of MHC class II reactivity *in vivo*, 500 μg of anti-MHC class II monoclonal antibody (clone M5/114 from BioXcell; LubioScience, Zurich, Switzerland) was administered intravenously 1 day before and 1 day after immunization (17).

### Analysis of Antigen-Specific CD8 T-Cell Responses by Flow Cytometry

Venous blood was collected and the erythrocytes were lysed (with Red Blood Cell Lysing buffer, Hybrid-Max, Sigma) before cell-surface staining was performed. Briefly, the cells were stained for 30 min with PE-labeled pentamer-OVA_257−264_ (5mer-SIINFEKL) from ProImmune (Oxford, United Kingdom) and fluorescently labeled antibodies specific for mouse CD3, CD4, CD8, CD44, and KLRG1 from eBiosciences (San Diego, CA, USA). Dead cells were excluded by staining with 7-Aminoactinomycin D (eBiosciences). All staining was performed on ice and protected from light.

Intracellular protein staining on erythrocyte-free blood cells or splenocytes was performed after overnight incubation of cells with 2 μM OVA aa257-264 peptide SIINFEKL (EMC microcollections; Tuebingen, Germany) in the presence of 7.5 μg/mL brefeldin A (Sigma-Aldrich). Briefly, the cells were first surface-stained with fluorescently labeled antibodies against mouse CD3, CD4, and CD8, and then fixed and permeabilized using Cytofix/Cytoperm and Perm/Wash solutions from BD Biosciences (San Diego, CA). Subsequently, the cells were stained with fluorescently labeled antibodies against IFN-γ, TNF-α, perforin, granzyme B, or FasL (eBiosciences) for 60 min. All stainings were performed on ice and protected from light. Finally, data was acquired on a BD Fortessa flow cytometer and analyzed with FlowJo software v.10 (Tree Star, Ashland, Oregon, USA).

### *In vivo* Cytotoxicity Assay

Splenocytes from naive CD45.1 mice were labeled with carboxy-fluorescein succinimidyl ester (CFSE) (Molecular Probes; Leiden, the Netherlands) at 5 μM (target population) or 0.5 μM (control population) according to the provider. The CFSE_hi_ target cells were pulsed with 0.5 μg/ml SIINFEKL peptide. After washing in PBS, the antigen-pulsed CFSE_hi_ target cells and the non-pulsed CFSE_lo_ control cells were mixed in a 1:1 number ratio and 100 μl administered intravenously into the previously immunized recipient C57BL/6 mice. Two days later, blood from these mice was collected, and the frequency of target cells was analyzed by flow cytometry. The percentage of specific killing was calculated based on the following equation:

(1) $$\begin{array}{r} SK=\left\{\;1-\frac{\left[\;\frac{CFSE\;high}{CFSE\;low\;}\;immunized\;mice\right]}{\left[\frac{CFSE\;high\;}{CFSE\;low}\;naive\;mice\right]}\;\right\}x\;100\;\% \end{array}$$

### Tumor Growth Experiment in Mice

To test the effect of PCI-based immunization on the growth of a mouse tumor, 2 ×10^5^ OVA-expressing B16 melanoma cells (B16-OVA) were injected subcutaneously into the flank of the mice. Tumor vaccination was first tested in a prophylactic setting where mice were immunized three times with 2 week intervals as described above (days 0, 14, 28) and then challenged with 2 ×10^5^ B16-OVA tumor cells by subcutaneous injection 1 week after the last vaccination (day 35). For therapeutic immunization, the mice were inoculated with 2 ×10^5^ B16-OVA tumor cells, immunized 7 days later with OVA and TPCS2a, and illuminated next day as described above. The immunizations were repeated weekly.

### Antibody Measurements

OVA-specific serum antibodies were analyzed in blood collected at different time points post immunization. Briefly, Maxisorb ELISA plates were coated with 4 μg/ml OVA and blocked with skimmed milk. Serial dilutions of mouse sera were applied and subsequently detected using biotinylated rat anti-mouse-IgG1 and IgG2c antibodies (BD Pharmingen). After ELISA development with strepatavidin-conjugated HRP (BD Pharmingen) and TMB substrate (eBioscience), absorbance was read at 450 nm using an ELISA plate reader from BioTek instruments (Sursee, Switzerland).

### Splenocyte Re-stimulation and Cytokine Secretion Assays

After PCI-based immunization with OVA, spleens were harvested, and the processed erythrocyte-free splenocytes were plated at 5 ×10^5^ cells per well in supplemented RPMI-1640 medium [with 8% FCS, 50 mg/ml Normocin (InvivoGen), 2 mM L-glutamine (Life Technologies, Zug, Switzerland), and 1 mg/mL G-418 (Life Technologies)] and incubated with 10 μg/ml OVA at 37 °C for 20 or 96 h. Cytokine secretion into the supernatant was measured using Ready-Set-GO! ELISA according to the manufacturer (Invitrogen by Thermo Fischer, Zug, Switzerland).

### Statistical Analysis

The statistical evaluations were performed using GraphPad Prism 7 (GraphPad Software). The data distribution was determined using a D'Agostino-Pearson normality test. If normally distributed, the data was further analyzed by student *t*-test or by two-way ANOVA. For non-parametric analysis, a two-sided Mann Whitney test or a Kruskal-Wallis test with Dunn's correction was applied. The significance of differences between the tumor survival curves was calculated with the log-rank (plotted curves with Kaplan-Meier) test using R (version 3.3.3; R Core Team, 2017). The significance level was set to 95%.

## Results

### PCI Facilitates Priming of Endogenous Antigen-Specific CD8 T Cells That Are Maintained Independent of MHC Class II Signaling

**Figure 1:**
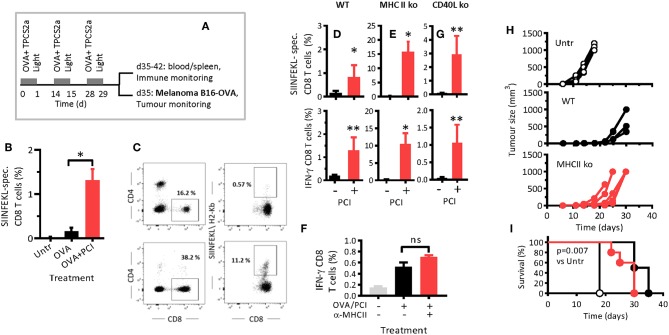
PCI facilitates CD8 T-cell proliferation and cytokine secretion independent of MHC class II and CD40L. **(A)** Schematic depiction of the experiment: Wild type C57BL/6 mice (WT) or syngeneic MHCII ko or CD40L ko mice were immunized with OVA or OVA combined with PCI and treated as illustrated in the scheme. **(B)** Antigen-specific CD8 T-cell responses in blood on day 35 following immunization in WT mice. **(C)** Expression of CD4, CD8, and SIINFEKL- specific (CD8) cells in WT and MHCII ko mice by flow cytometry. **(D,E,G)** Frequencies of SIINFEKL-specific CD8 T cells in blood on day 35 in WT, MHCII ko, and CD40L ko mice, and intracellular IFN-γ analysis after *ex vivo* re-stimulation of blood cells from immunized WT **(D)**, MHCII ko **(E)**, and CD40L ko **(G)** mice. **(F)** WT mice were treated with MHCII-blocking antibodies and immunized as above with OVA and PCI. SIINFEKL-specific IFN-γ production was measured in spleen cells after *ex vivo* re-stimulation. **(H)** Groups of 5 WT and MHCII ko mice were immunized thrice with OVA and PCI and challenged with 2 ×10^5^ B16-OVA melanoma cells subcutaneously. Tumor growth in individual mice **(H)** and Kaplan Meier survival plots **(I)** are shown. *p ≤ 0.05, **p < 0.01 calculated by non-parametric Mann-Whitney test. The experiments in WT and MHCII ko mice were performed at least three times with comparable results. The experiment in CD40L mice was performed twice. Shown are means + SEM from one representative (*n* = 5 mice per group). The tumor challenge experiment was performed twice with comparable results. *P* = 0.007 comparing non-immunized WT mice (Untr) to immunized WT or MHCII ko mice and evaluated with the log-rank test of the Kaplan-Meier curves.

Wild type (WT) mice were immunized thrice with OVA or OVA combined with PCI (Figure 1A), and antigen-specific CD8 T-cell proliferation and cytokine secretion were analyzed by flow cytometry (Supplemental Figure 1). One week after the last immunization (day 35), the frequency of antigen-specific CD8 T cells in blood had risen from 0.02% in non-immunized mice to 0.15% in OVA-immunized mice (Figure 1B). In mice that also received PCI treatment, a further 10-fold increase (1.25%) was detected. Hence, PCI-supported immunization can facilitate activation and proliferation of CD8 T cells, in WT mice.

The hypothesized mechanism of PCI-based immunization is the endosomal escape, cytosolic release, and MHC class I presentation of processed antigen to CD8 T cell. These events are supposed to be triggered by light activation of photosensitizer contained in DC endosomes and cause a diversion of the antigen away from MHC class II presentation. Hence, we investigated if host MHC class II molecules were required in intradermal OVA immunization as a function of PCI support. MHC class II-deficient (MHCII ko) mice were immunized with OVA with or without PCI. The MHCII ko mice were expectedly lacking CD4 T cells (Figure 1C). Immunization with OVA alone resulted in weak antigen-specific CD8 T-cell proliferation in WT mice (Figure 1D), and no measurable response in MHCII ko mice (Figure 1E). Surprisingly, activation and proliferation CD8 T-cells were not impaired in MHCII ko mice when OVA immunization was combined with PCI (Figure 1E). Indeed, the measured frequencies of antigen-specific CD8 T cells in blood from MHCII ko were 15% (Figure 1E) while typically 1–5% in WT mice (Figure 1D and data not shown from replica experiments). In addition, the capability of the CD8 T cells to produce interferon-γ (IFN-γ) was maintained independently of MHCII for PCI-based immunization (Figures 1D,E). Since MHCII ko mice were deficient both in MHCII and in CD4 T cells, and to test if the PCI-associated amplification of CD8 T-cell responses in MHCII ko mice was associated with the lack of MHCII or the lack of CD4 T cells, WT mice were treated with anti-MHC class II antibody and immunized. The blocking of MHC class II did not affect CD8 T-cell activation and IFN-γ production (Figure 1F). Hence, PCI- based immunization enabled the priming and maintenance of endogenous antigen- specific CD8 T cells, independently of MHC class II signaling. Of note, the PCI-facilitated activation of CD8 T cells was dependent on light-activation of the photosensitizer, since immunization with antigen and TPCS2a without application of light did not trigger antigen-specific CD8 T-cell proliferation and cytokine secretion for OVA (Supplemental Figure 2) or for other antigens (unpublished data).

### PCI Facilitates CD8 T Cell-Priming Independent of CD40-CD40L Signaling

CD40L on CD4 T cells may interact with CD40 on APCs and thereby provide co-stimulatory signals that help effective priming of CD8 T cells. Since PCI enabled CD8 T-cell activation in the absence of CD4 T cells, we tested whether CD40 signaling was dispensable in PCI-based immunization. To this end, CD40L-deficient mice were immunized. Neither proliferation nor IFN-γ production (Figure 1G) of SIINFEKL-specific CD8 T cells depended on CD40-signaling when immunization was combined with PCI. In immunized mice that were not PCI treated, the responses in WT and CD40L ko mice were comparably low. These results suggest that the PCI-facilitated priming of antigen-specific CD8 T cells takes place independent of CD40.

### PCI-Based Vaccination Prevents Subsequent Growth of Mouse Melanoma Independent of MHC Class II Signaling

PCI-based vaccination and the role of T helper cells and MHCII signaling in PCI-based vaccination was further studied in a mouse tumor model. WT and MHC class II-deficient mice were treated fortnightly with OVA protein and by PCI as described above (Figure 1A), and 1 week after the last booster, the mice were challenged with B16-OVA cells. Tumor growth (Figure 1H) and survival (Figure 1I) were significantly delayed in vaccinated mice as compared to non-vaccinated mice, independent of MHC class II. The mean survival times for untreated, WT, and MHCII ko mice were 18, 33, and 30 days, respectively.

### CD8 T-Cell Activation Is Not Impaired, but Long-Lasting After CD4 T-Cell Depletion

**Figure 2:**
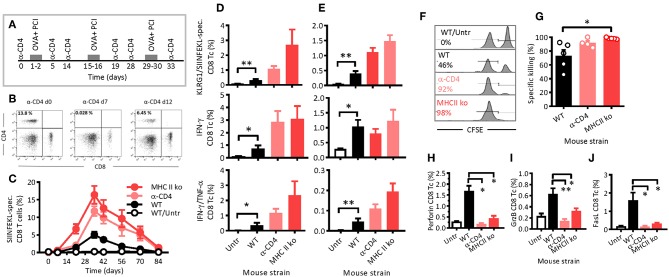
PCI-mediated immune responses are long lasting, independent of CD4 T-helper cells and cytotoxic. **(A)** Schematic depiction of the experiment: WT or MHCII ko mice were immunized thrice with OVA and PCI. Additionally, WT mice were treated with CD4-depleting antibodies, whereas complete untreated mice were also included. **(B)** The effects of anti- CD4 depletion monitored by flow cytometry prior to treatment on day 7 and 12. **(C)** Kinetics of SIINFELK-specific CD8 T cells in blood was monitored by flow cytometry. Blood cells **(D)** and spleen cells **(E)** were analyzed eight **(D)** and nine **(E)** weeks after the last immunization for frequencies of SIINFEKL-specific and KLRG1-expressing CD8 and CD44-positive lymphocytes, intracellular IFN-γ-production, IFN-γ, and TNF-α double-producing CD8 T cells. **(F–H)** Mice were immunized as shown in the scheme **(A)**, and cytotoxicity **(F,G)** was measured *in vivo* with SIINFEKL-loaded and CFSE loaded spleen cells from CD45.1 mice. The mean percentages of the killing activity are presented based on the frequency of target cells detected in blood. **(F)** Representative flow-cytometry histograms of SIINFEKL-loaded CFSE_high_ target cells (right peak) and CFSE_low_ control cells (left peak). **(G)** Summary plot. **(H–J)** Seven days after the target-cell transfer, CD8 T cells present in spleen of the immunized mice were analyzed for the expression of perforin **(H)**, granzyme B **(I)**, and FasL **(J)**. The experiments were performed at least twice with comparable results. *p ≤ 0.05 and **p < 0.01 analyzed with Kruskal-Wallis. Shown are means + SEM (*n* = 5 mice per group).

To further study how CD4 T cells affected PCI-based immunization during antigen presentation, we removed CD4 T cells in WT mice by intraperitoneal injections of CD4 T-cell depleting antibodies 1 day prior to and 4 days after each of the three immunizations (Figure 2A). The CD4 T- cell depletion was complete within 1 day of antibody injection, lasted for at least 2 days after the last antibody injection (Figure 2B), and produced a CD4 phenotype similar to that of MHCII ko mice, which were used as CD4-deficient control (Figure 1C). By day 12, the CD4 T-cell population largely recovered in mice receiving depleting antibodies.

Typically, antigen-specific CD8 T cells were not observed after the primary immunization, but were clearly detectable upon a first booster injection, especially in MHCII ko mice, but also in WT and CD4 T-cell depleted mice (Figure 2C). Maximum frequencies of SIINFEKL-specific CD8 T cells in blood were determined 1 week after the second booster, and were 15, 12, and 5% for MHCII ko, CD4 T-cell-depleted, and WT mice, respectively. Thereafter, the frequencies decreased comparably, independent of CD4 T cells or MHC class II. Hence, neither MHC class II signaling nor CD4 T-helper cells were required for PCI-mediated stimulation of CD8 T-cell responses, and the two tested models of CD4 T-cell deficiency were comparable. On week 12, i.e., 8 weeks after the last booster, SIINFEKL-specific CD8 T cells were comparable through all groups with frequencies close to baseline levels. Here, blood (Figure 2D) and spleen (Figure 2E) cells were isolated and re-stimulated *in vitro* with SIINFEKL. In general, lymphocytes from immunized WT mice revealed significantly stronger recall effects than cells from untreated mice as measured by the expression of KLRG1 and the secretion of IFN-γ and TNF-α cytokines. Hence, the results suggest that PCI-based immunization triggered functional CD8 T-cell memory. Moreover, the functional memory was independent of CD4 T cells or MHC class II, since KLRG1 expression and cytokine secretions were not reduced in MHCII ko and CD4 T-cell depleted mice as compared with WT mice.

The depletion of CD4 T-cells by injection of depleting antibodies 4 days after each immunization had no effect on the CD8 T-cell activation when compared with the WT mice (data not shown). In order to test if the WT CD4 T-cell population contained an immune suppressive factor that negatively affected the CTL activation, MHCII ko mice received an adoptive transfer of purified WT CD4 T cells or Foxp3- and CD25- CD4 regulatory T cells (Tregs) prior to the PCI-based immunization. No CTL-specific immunosuppressive effects of the transfer were observed (Supplemental Figure 3). Therefore, with these experiments, we confirmed activation and long-lasting functions of CD8 T cells in mice even in the absence of CD4 T-helper cells.

### Cytotoxicity Upon PCI-Based Immunization Is Not Dependent on MHC Class II or CD4 T- Cell Help

Assessment of *in vivo* cytotoxicity showed that PCI-based immunization also induced cytotoxic functions, and again, the property was not dependent on CD4 T cells or MHC class II signaling (Figure 2F). In fact, the measured cytotoxicity was nearly 100% in immunized MHC class II-deficient and in CD4 T-cell-depleted mice, while only 73% in immunized WT mice (Figure 2G). In contrast, the expression of cytolytic mediators such as perforin, granzyme B, and FasL was upregulated on CD8 T cells from WT mice, but not on CD8 T cells from MHC class II-deficient and from CD4 T-cell-depleted mice (Figures 2H–J). Hence, PCI-based immunization mediates CD4 T-cell-independent cytotoxicity, but it seemed that the induced expression of the cytolytic proteins perforin, granzyme B, and FasL depended on co-stimulatory or bystander signals from T-helper cells.

### Therapeutic PCI-Based Vaccination Suppressed Growth of Melanoma Independent of CD4 T Cells

**Figure 3:**
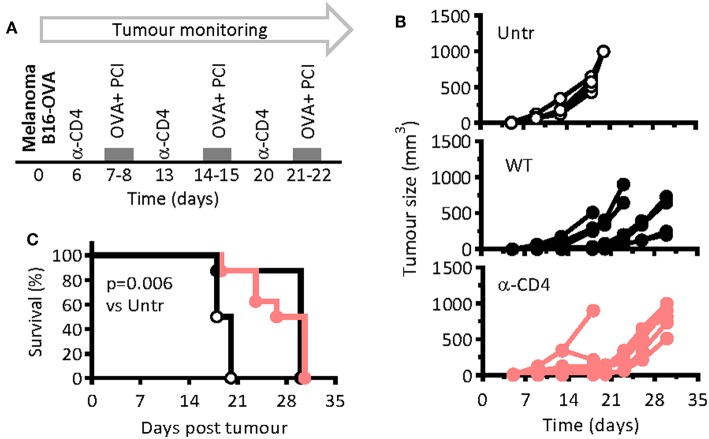
PCI-mediated immunization prevents melanoma growth in mice independent on CD4 T-cell help. **(A)** WT mice were injected subcutaneously with 2 ×10^5^ B16-OVA melanoma cells and were therapeutically immunized weekly with OVA and PCI as indicated in the scheme. One group of mice was additionally treated with CD4 T-cell depleting antibodies on days 6, 13, and 20. **(B)** Tumor growth in individual mice (*n* = 4–8 mice per group). **(C)** Kaplan Meier survival plots. *p* < 0.001 and evaluated with the log-rank of the Kaplan-Meier curve. The experiments were performed twice with comparable results.

PCI-based CTL vaccination was also tested in a therapeutic setting of tumor-bearing mice, including that of CD4 T-cell-depleted mice. Seven days after the inoculation of the melanoma cells, at which time point a solid tumor was visible palpable, the mice were vaccinated and PCI treated with weekly intervals (Figure 3A). For depletion of CD4 T cells, anti-CD4-depleting antibodies were administered 1 day prior to each vaccination session. The tumor grew fast in non-vaccinated control mice, while the growth was delayed in vaccinated mice (Figure 3B). The lack of CD4 T cells did not notably impair anti-tumor immune responses in immunized mice, the melanoma growth and mouse survival being comparable in WT and in CD4 T-cell-depleted mice (Figure 3C). The mean survival time for untreated, WT, and CD4-depleted mice were 19, 28, and 30 days, respectively, pointing out the therapeutic effect of PCI-based vaccination against melanoma, independent of CD4 T cells.

### PCI-Based Vaccination With Melanoma-Associated Antigens Is Also Independent of T-Cell Help

**Figure 4:**
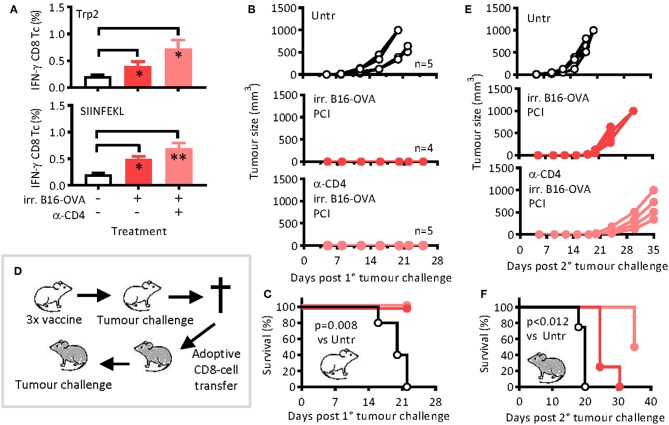
Antigen-specific CD8 T-cell responses and tumor remission after immunization with irradiated melanoma cells and PCI treatment. Mice were immunized on days 0, 14, and 28 with irradiated B16-OVA melanoma cells ± PCI. **(A)** On day 21, IFN-γ cytokine production was measured in blood cells after re-stimulation with melanoma Trp2 (top) or SIINFEKL (bottom) peptides. Shown are means + SEM (*n* = 4–5 mice per group). On day 29, mice were challenged with 2 ×10^5^ B16-OVA cells subcutaneously and **(B)** tumor growth and **(C)** survival were monitored. The *p* value (*p* = 0.008) was calculated by log-rank analysis of the Kaplan-Meier curves comparing non-immunized mice with any of the two immunized groups. **(D)** On day 22 after tumor inoculation, mice were euthanized, and CD8 T cells were MACS-sorted and adoptively transferred into groups of four naïve WT mice (1.5 ×10^7^ cells/mouse). The recipient mice were then challenged with B16-OVA cells as above. **(E)** Tumor growth and **(F)** survival were monitored. *p ≤ 0.05 and **p < 0.01 analyzed with Kruskal-Wallis. Pairwise comparison indicate that transfer of immune CD8 T cells caused significant survival benefit over the transfer of naïve CD8 T cells (*p* < 0.012) when calculated by log-rank analysis of the Kaplan-Meier curves. The experiments were performed twice with comparable results.

To test the added immunological effect of PCI on cancer vaccination on other antigens than OVA, we immunized mice as for OVA but using irradiated B16-OVA melanoma cells as antigen. The cells naturally express known melanoma antigens such as gp100 and Trp2 but were engineered to also express OVA. Again, the vaccination was followed 1 day later by PCI treatment, and one group of mice was treated with CD4-depleting antibodies prior to each vaccination session. After re-stimulation of blood lymphocytes *ex vivo* with melanoma Trp2 or OVA peptides, significant CD8 T-cell responses against both antigens could be detected, and the responses were not dependent on CD4 T-cell help (Figure 4A). One day after the last of three vaccinations (day 29), all mice were challenged with live melanoma cells and the tumor growth monitored. None of the immunized mice showed tumor growth (Figure 4B), and the mice were protected beyond day 22 post-tumor challenge independently of the presence of CD4 T-helper cells (Figure 4C). On day 22 after the tumor challenge, the mice were euthanized, the purified CD8 T cells from spleens and lymph nodes were adoptively transferred into naïve WT hosts, and the hosts were then challenged with live melanoma cells to test the fitness of non-helped CD8 T cells (Figure 4D). Control mice were adoptively transferred with purified CD8 T cells from naïve mice. Tumor growth was observed in all mice, but the growth was delayed in mice that had received CD8 T cells from immunized mice as compared to the mice that had received naïve CD8 T cells (Figure 4E). Moreover, the melanoma growth was also delayed in mice that had received non-helped CD8 T cells from immunized CD4-T-cell-depleted mice. The Kaplan Meier curves (Figure 4F) revealed a median survival time of 20 days of the mice that had received naïve CD8 T cells (“Untr”) and a survival benefit for all immunized mice (*p* = 0.012). For mice that received immune CD8 T cells, there was a survival benefit for non-helped CD8 T cells over mice that received helped CD8 T cells (*p* = 0.012), the median survival times being 35 and 27 days, respectively. As for the model protein OVA, potential therapeutic effect of PCI-based vaccination was also observed with tumor-relevant antigens. Moreover, the results also suggest that the PCI method of vaccination is also applicable to cellular vaccines and not only to the so-far tested soluble protein vaccines.

### Increased CD8 T-Cell Responses by PCI Did Not Impair CD4 T-Cell Dependent Antibody Responses

**Figure 5:**
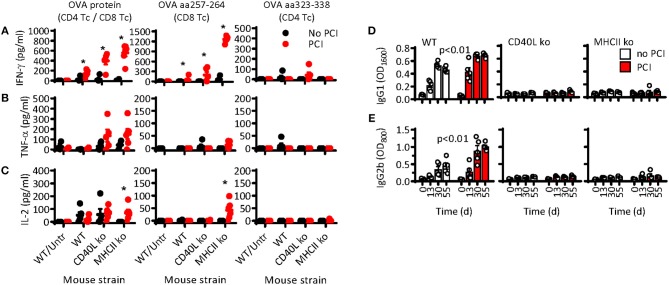
CD8 T-cell-mediated cytokine secretion but not B-cell-mediated antibody production is independent of T helper cells upon PCI-based immunization. Wild type C57BL/6 mice (WT) or syngeneic MHCII ko or CD40L ko mice were immunized on day 0, 14, and 31 with OVA (black symbols and white bars) or OVA combined with PCI (red symbols and bars). The mice were bled for analysis of antibodies on days 0, 13, 30, and 55. **(A–C)** Spleen cells were harvested on day 55 and re-stimulated *in vitro* with OVA protein (*left panel*), MHCI-binding OVA aa257-264 (*middle panel*), or MHCII-binding OVA aa323-338 (*right panel*). The supernatants were harvested after 72 h (**A**: IFN-γ and **B**: TNF-α) or 24 h (**C**: IL-2) and the cytokine secretion analyzed by ELISA; *p ≤ 0.05 analyzed with Mann Whitney and comparing PCI vs. non-PCI within each strain. The blood serum was analyzed on day 0, 13, 30, and 55 for OVA-specific **(D)** IgG1 and **(E)** IgG2b by ELISA. Results are expressed as optical density at a serum dilution of 1:1,600 for IgG1 and 1:800 for IgG2b and analyzed by two-way ANOVA comparing PCI vs. non-PCI within each strain.

While PCI-based immunization clearly facilitated CD8 T-cell responses independent of helper T cells, it is thus far unclear whether PCI also aids the stimulation of CD4 T-cell responses or T-cell mediated B-cell responses. To address this question, WT, CD40L ko, and MHCII ko mice were immunized thrice with OVA or with OVA combined with PCI, as described above (cf. Figure 1A). Two weeks after the third immunization, spleens were harvested and the cells re-stimulated *in vitro* with OVA protein, MHCI-binding OT-I peptide (OVA aa257-264: SIINFEKL), or MHCII-binding OT-II peptide (OVA aa323-338). Re-stimulation with OVA protein enabled us to study mixed CD4 and CD8 T-cell responses, while OT-I and OT-II peptides stimulate specific CD8 and CD4 T-cell subsets, respectively. The cell culture supernatants were assessed for IFN-γ, TNF-α, or IL-2 cytokines. OVA protein caused a strong secretion of IFN-γ in cells from mice immunized with PCI support (Figure 5A). As observed by flow cytometry above (cf. Figures 1D–F), the IFN-γ secretion was particularly strong in MHCII- and CD40L-deficient cells. The IFN-γ was primarily produced by OT-I-reactive CD8 T cells, as re-stimulation with OT-I and OVA protein produced similar levels of IFN-γ. For cells stimulated with OT-II peptide, little IFN-γ secretion was measured and only in cells from CD40L ko mice that were immunized with PCI support. Significant release of TNF-α was also observed in CD40L- and MHCII-deficient cells after stimulation with OVA, and the effect was facilitated by PCI treatment (Figure 5B). OVA-specific IL-2 secretion was comparable in cells from immunized WT or CD40L ko mice, independent of PCI treatment (Figure 5C). As for IFN-γ secretion, the TNF-α and especially the IL-2 secretion was SIINFEKL specific and highly elevated in cells from PCI-treated MHCII ko mice. No OT-II-specific TNF-α and IL-2 secretion were observed.

Since CD4 T-cell responses were almost undetectable, we also analyzed mouse sera for OVA-specific antibodies, which are expected to be CD4 T-cell dependent due to the requirement for CD4 T-helper cells for B-cell activation. Prior to and 14 days after each immunization, the mice were bled for analysis of OVA-specific antibodies in serum. Both IgG1 (Figure 5D) and IgG2b (Figure 5E) were induced in WT mice, independently of the PCI treatment. In contrast, no IgG3 antibody responses were observed (data not shown), an isotype that is often induced by T-cell independent antigens (*p* < 0.01 by two-way ANOVA). The igG1 and IgG2b antibody responses were slightly higher in PCI-treated mice than in mice that received OVA immunization only. A sero-conversion was observed on day 13, hence after a single immunization, and the response plateaued after the second immunization. No OVA-specific antibody response was observed in CD40L ko and in MHCII ko mice.

## Discussion

Cancer vaccines can trigger endogenous immune responses to autologous tumors, including such leading to tumor remission. Typically, the protective cellular responses have been ascribed to CD8 T cells, since tumor cells express little, if any, MHC class II molecules (18). Unfortunately, the therapeutic benefits of cancer vaccines are still ambiguous, in part due to immunosuppressive signals produced by the tumors, which also include the down regulation of MHC class I molecules (19). Hence, it is important to develop strategies that trigger strong tumor-specific cytotoxic immune responses, while overcoming potentially immunosuppressive tumor microenvironments.

While it is well recognized that CD8-positive CTLs are important players in the immune surveillance of tumors, there is an ongoing controversy concerning the importance of CD4 T-cell help for the induction of CTLs (20–24). Antigen-specific CD4 T cells can improve expansion of cognate and tumor-specific memory CD8 T cells (25), enhance recruitment and infiltration of CD8 T cells into tumors (26), help overcoming immunosuppressive reactions in the tumor microenvironment (27), and facilitate adoptive CD8 T cell tumor therapy (28). In one study, tumor-specific CD8 T cells were activated independently of CD4 T cells, but required Th1-polarized CD4 T cells for effective tumor suppression (29). Similar CD4 T-helper support functions have been reported in viral infections or anti-viral vaccination (30). Nonetheless, other studies concluded that T-helper cells are required merely for the priming of CTLs cells (31), or for the maintenance of CD8 T-cell memory (22, 32). Yet, functional CD8 T-cell responses were also shown to develop without contribution of CD4 T cells (33–35), the help in part being replaced by signals provided through activation of pattern recognition receptors such as Toll-like receptors (36). Since cancer vaccination aims at triggering cytotoxic and tumor-specific CTLs, it is important to understand how CD4 T-helper cells may contribute to CTL priming, function and survival in the context of cancer vaccines.

Most recently, vaccination and parallel photochemical treatment, so-called PCI-based vaccination, was described as a novel vaccination method that can trigger CTLs, at least *in vitro* and in transgenic mice using OVA as model antigen (12, 13, 15). The current study builds on these studies with two objectives. Firstly, does PCI facilitate CD8 T-cell activation *in vivo* in wild type mice? Secondly, how are the T-helper requirements in such an experimental setting, bearing in mind that PCI is expected to cause translocation of antigen away from the default MHC class II pathway of antigen presentation?

The data generated in the current study fully supported previous *in vitro* data and data using transgenic mice recognizing OVA antigen. We primarily applied OVA in the current study, but we could also demonstrate that PCI-based vaccination using lethally irradiated melanoma cells caused activation of melanoma specific CD8 T-cell responses. Hence, the data represents a proof of principle and provide arguments to take the technology a step further toward testing in other pre-clinical models or even clinical trials. Indeed, a phase I trial is currently performed to investigate the safety of intradermal administration of photosensitizer and subsequent light treatment (ClinicalTrials.gov Identifier: NCT02947854).

Regarding the second research objective, the hypothesized mechanism of action of PCI-based vaccination included the diversion of antigen from default MHC class II- toward a MHC class I-pathway of antigen presentation. Indeed, PCI facilitated a shift in the immune response from MHC class II-restricted CD4 T-cell and B-cell responses toward CD8 T-cell responses (12, 13). However, if priming of CTLs depends on CD4 T-helper cells, the obvious question is how bypassing MHC class II affects CTL activation. The current work suggests that PCI-based vaccination elicits strong and functional CD8 T-cell responses, independently of help from CD4 T cells. The study also suggest that PCI-based vaccination may be considered a treatment option in cancer immunotherapy alongside other new vaccination strategies and immunotherapies. While previous studies used T-cell-receptor transgenic mouse models and the model antigen OVA to proof the concept of PCI-based CTL vaccination (12, 13, 15), the current study showed vaccine efficacy in WT mice, using different antigens (OVA and melanoma antigens), as well as different antigen vehicles (soluble protein and irradiated tumor cells).

The observed quality of the anti-tumor and CD8 T-cell mediated immune responses in MHC class II-deficient mice was both striking and surprising. The fact that naïve MHCII ko mice have a slightly higher number of CD8 T cells than the WT mice can only partly explain the result. Moreover, the improved CTL activation in MHCII ko mice did not appear to be a result of the lack of MHC class II expression *per se*, as treatment of WT mice with anti-MHC class II antibodies did not affect CTL activation. It was rather the lack of CD4 T cells in MHCII ko mice that mediated the favorable CTL effect, because transient depletion of CD4 T cells using CD4 T-cell depleting antibodies strongly facilitated CD8 T-cell responses as well as tumor remission. Of note, the “beneficial” effect of lacking CD4 T cells seemed to be mediated by a mechanism active during antigen presentation and not by a later and general bystander effect of CD4 T cells. When CD4 T cells were depleted a few days after the vaccine administration, no improved effect of CD8 T-cell activation was observed.

Hence, how could the lack of CD4 T cells be beneficial for CTL activation, or how can CD4 T cells prevent optimal CTL activation? Numerous studies have demonstrated the potential immune suppressive mechanisms of regulatory CD4 T cells (Tregs) (37). Through ligation with the co-stimulatory molecules CD80 and CD86 molecules on the surface APCs, the inhibitory receptor CTLA-4 is a potent mediator of Treg-induced immunosuppression (38). The impact of preventing CTLA-4-mediated suppression has become evident in melanoma patients where the blocking of this checkpoint has improved treatment success and life expectancy (39). The depletion of Tregs has also been shown to delay tumor growth upon vaccination in melanoma-bearing mice (40), while depletion of the whole CD4 T-cell population showed no such effect (35). Co-stimulatory signals to CD8 T cells can also be transferred via CD27-CD70 interactions (36, 41), and Tregs have been shown to inhibit anti-tumor CTLs, in part by an enhanced CD70 sensitivity (42). The CD27 receptor is expressed on CD4 T cells, naïve CD8 T cells, natural-killer cells, and B cells, while the CD70 ligand is upregulated on activated DCs and along with CD80 and CD86. However, the expression of CD70 on DCs was not reduced in CD4-depleted mice, and CD27 was essential for the generation of helped CTLs and for their secondary expansion. Since the exact mechanism by which PCI improves CTL vaccination is not known, we speculated that the absence of Tregs in MHCII- and CD4 T-cell-deficient mice could be contributing to the improved CD8 T-cell responses that we observed upon PCI-based vaccination, but not upon conventional vaccination. We tested this by using an adoptive transfer of purified WT CD4 T cells or Tregs into MHCII ko mice prior to their immunization. However, the CD8 T-cell responses remained high in the adoptively transferred MHCII mice. Therefore, the facilitated CD8 T-cell responses in MHCII- and CD4 T-cell-deficient mice upon PCI-based immunization is unlikely caused by the lack of potentially immune-suppressing Tregs.

In order to gain insight into the role of CD4 T cells on CTL activation, we studied the pathways by which CTL responses are triggered. One of the principal mechanisms by which CD4 T cells help the CTL cells priming is via licensing of DCs through the CD40-CD40L interactions, leading to maturation of DCs prior to their priming of CD8 T cells (43). In the absence of CD4 T cells, CD40 signaling can substitute the otherwise required T-helper signals, and the administration of agonistic anti-CD40 antibodies can improve vaccine efficacy in CD4 T-cell deficient conditions (34). Moreover, a subset of CD8 T cells can express CD40L allowing an autocrine activation of CD8 T cells independent of T-helper cells (43). In the current study, we showed that PCI-based vaccination was CD4 T-cell independent, but that the T-helper signals were not substituted by CD40 signals, since CD8 T-cell responses were not impaired in CD40L-deficient mice.

The above-mentioned findings suggest that, not CD40 but another signaling pathway, may provide the co-stimulation and activation of CD8 T cells when immunizing with PCI in the absence of MHCII or CD4 T cells. To this end, T-helper signals have also been shown to be bypassed with innate danger signals, e.g., TLR signals and pro-inflammatory cytokines that can directly activate APCs for cross-priming of antigen to CD8 T cells (44). In the absence of T-helper cells, CTLs could be induced when short MHC class I-binding peptides were co-administered with TLR3 and TLR9 ligands, but not with TLR2, TLR4, TLR6, or TLR7 ligands (45). In the current study, although we did not use any adjuvant combined with the PCI treatment, we cannot exclude the possibility that the photochemical reaction induced by PCI induces danger signals that eventually act on inflammatory pathways through up-regulation of cytokines and other inflammatory proteins. Although, PCI-based vaccination in MyD88- or TRIF-deficient mice did not suggest a PCI-mediated TLR-effect (unpublished data), it will be important to investigate the inflammatory reactions induced by PCI and how these affect antigen presentation. A recent study on using PCI for peptide vaccination shows that *in vitro* PCI can increase CD86 expression in APCs, indicating that the photochemical treatment may have an inherent adjuvant activity (46).

Since PCI-based vaccination is applied intradermally, dermal DCs at the injection site are likely the targeted cells. Although, the exact subsets of DCs involved and the duration of such activation remain unknown, pro-inflammatory cytokines can directly activate APCs for cross-priming of antigen to CD8 T cells (44). Clinical data revealed that elevated IFN-γ expression in the tumor is associated with improved survival in melanoma (47). IFN-γ can also mediate upregulation of MHC class I and class II molecules and of antigen processing and presentation, which again may enhance recognition of tumor-associated antigens by T cells (48). The migration of T cells from lymph nodes to the tumor is also induced by IFN-γ and the cytokine can induce expression of the IFN-responsive chemokines (49). In the current study, PCI especially facilitated strong IFN-γ production by CD8 T cells and one may speculate that this property may contribute to the T-helper cell independency.

One possible pitfall of a vaccination method that facilitates MHC class I-restricted antigen presentation concerns the potential activation of autoimmune responses. Normally, the presentation of self-antigens takes place in the absence of inflammation, leading to anergy, or apoptosis of the T cells. However, since PCI is followed by local inflammation, the APCs may be equipped with co-stimulatory molecules, a process that in the end may lead to a break of peripheral tolerance. We have not observed signs of autoimmune responses with PCI, but such processes are typically slow, for which reason long-term studies would need to be performed. On the other hand, since PCI-based vaccination would be applied to cancer patients, a risks-benefit assessment would anyhow be required. However, the currently and widely applied immunotherapies with checkpoint inhibitors similarly increases the risk of immune-related adverse events, including a potential shift from self-tolerance to autoimmunity. Hence, this potential adverse event is not only an attribute to PCI-based vaccination. Nonetheless, this is a topic that must be kept in mind in future studies of PCI-based vaccination.

In closing, the presented data clearly demonstrates that PCI-based vaccination is an effective method for stimulation of tumor-specific CTLs, and that these CD8 T cells can suppress tumor growth. Interestingly, the mechanism by which PCI-based vaccination exerted its action was strictly independent of CD4 T-helper cells, at least in mice. Even in the absence of such bystander help, the CTLs had effector and memory phenotypes similar to wild type immune responses and prevented melanoma growth in mice, both in prophylactic and therapeutic setting. Although the exact mechanism by which PCI circumvents the need of CD4 T-cell help in vaccination is not known, this feature could be of therapeutic relevance. It shows that therapeutic vaccination may be feasible in situations where patients manifest with CD4 T-cell deficiency, for instance due to disease or medical immune suppression. At present, this new method of CTL vaccination is also validated in a first clinical human trial, the primary objective being the incidences of adverse events after a single administration of photosensitizer and light, but immune response to co-administered human papilloma virus E7 (HPV E7) and keyhole limpet hemocyanin (KLH) antigens will be assessed in blood (NCT02947854).

## Data Availability

The datasets generated for this study are available on request to the corresponding author.

## Ethics Statement

All mice were kept under SPF conditions, and the experimental procedures were approved by the animal research ethics review board and the Swiss veterinary authorities (licenses ZH 200/2014 and ZH 52/2016).

## Author Contributions

EV, FH, and YW-M performed experiments. EV, AH, BG, CH, and PJ designed experiments. TK provided resources. EV and PJ wrote the manuscript. All authors reviewed the manuscript.

### Conflict of Interest Statement

AH is employee of PCI Biotech, which has field patents on the use of photosensitizer in vaccination. AH also own shares in PCI Biotech. AH and PJ are mentioned as inventors of patents describing the use of PCI in immunization and vaccination. The remaining authors declare that the research was conducted in the absence of any commercial or financial relationships that could be construed as a potential conflict of interest.
