# Supplementary material for: Combined Photosensitization and Vaccination Enable CD8 T-Cell Immunity and Tumor Suppression Independent of CD4 T-Cell Help
Source: Front Immunol. 2019 Jul 5;10:1548. doi: 10.3389/fimmu.2019.01548 (PMC6624637; doi:10.3389/fimmu.2019.01548)
Supplement: Supplementary file 1 [file Table_1.DOCX]

# Supplementary Materials

Eleni Maria Varypataki et al.

Combined photosensitization and vaccination enable CD8 T-cell immunity and tumor suppression independent of CD4 T-cell help

A


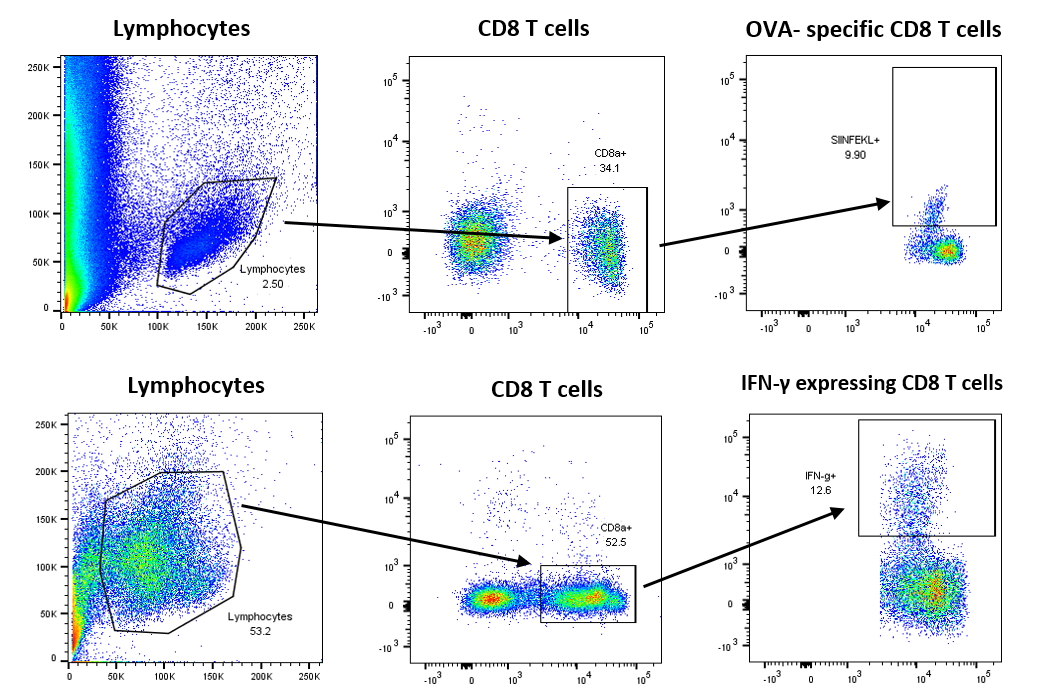


**FSC**

**CD8**

**IFNγ**

**CD4**

**CD4**

**CD8**

**FSC**

**SSC**

**SSC**

**Pentamer**

**CD8**

**CD8**

**12.6 %**

**52.5 %**

**9.90 %**

**34.1 %**

B

### Supplemental Figure S1

Representative flow cytometry gating strategy for detection of SIINFEKL- specific CD8 T cells (**A**) and IFN-γ producing CD8 T cells (**B**) in immunized MHC II ko mice at the time of the blood analysis presented in Figure 1D-E.

### Supplemental Figure S2

Experiment testing the adjuvant effect of light or TPCS2a alone. Mice were immunized intradermally with OVA with or without TPCS2a as described above. Light was applied for 6 minutes 18 hours after the injections. Twelve days after last injection, spleens were harvested for assessment of SIIFNKEL-specific CD8 T cells (*left*) or IFN-γ producing CD8 T cells (*right*).

|  |  |
| --- | --- |
|  |  |

### Supplemental Figure S3

PCI-Induced CD8 T-cell-responses monitored in spleens of WT, MHC class II ko mice, and in MHC class II ko mice that were intravenously injected with purified naïve MACS-sorted CD4 T cells or with purified MACS-sorted CD25-positive CD4 cells (Treg) prior to immunization. Mice shown were analyzed with SIINFEKL pentamers for antigen-specific responses (*top left*), expression of the memory marker KLRG1 in the SIINFEKL-specific population (top right), intracellular production of IFN-γ (*bottom left*), and intracellular production of both IFN-γ and TNF-α (*bottom right*) by CD8 T cells.
